# Memristive Non-Volatile Memory Based on Graphene Materials

**DOI:** 10.3390/mi11040341

**Published:** 2020-03-25

**Authors:** Zongjie Shen, Chun Zhao, Yanfei Qi, Ivona Z. Mitrovic, Li Yang, Jiacheng Wen, Yanbo Huang, Puzhuo Li, Cezhou Zhao

**Affiliations:** 1 Department of Electrical and Electronic Engineering, Xi’an Jiaotong–Liverpool University, Suzhou 215123, China; Zongjie.Shen@xjtlu.edu.cn (Z.S.); Yanfei.Qi01@xjtlu.edu.cn (Y.Q.); Jiacheng.Wen16@student.xjtlu.edu.cn (J.W.); Yanbo.Huang16@student.xjtlu.edu.cn (Y.H.); Puzhuo.Li19@student.xjtlu.edu.cn (P.L.);; 2 Department of Electrical Engineering and Electronics, University of Liverpool, Liverpool L69 3BX, UK; Ivona@liverpool.ac.uk; 3 School of Electronic and Information Engineering, Xi’an Jiaotong University, Xi’an 710061, China; 4 Department of Chemistry, Xi’an Jiaotong–Liverpool University, Suzhou 215123, China; Li.Yang@xjtlu.edu.cn; 5 Department of Chemistry, University of Liverpool, Liverpool L69 3BX, UK

**Keywords:** non-volatile, memristor, graphene-based materials, operation mechanisms

## Abstract

Resistive random access memory (RRAM), which is considered as one of the most promising next-generation non-volatile memory (NVM) devices and a representative of memristor technologies, demonstrated great potential in acting as an artificial synapse in the industry of neuromorphic systems and artificial intelligence (AI), due its advantages such as fast operation speed, low power consumption, and high device density. Graphene and related materials (GRMs), especially graphene oxide (GO), acting as active materials for RRAM devices, are considered as a promising alternative to other materials including metal oxides and perovskite materials. Herein, an overview of GRM-based RRAM devices is provided, with discussion about the properties of GRMs, main operation mechanisms for resistive switching (RS) behavior, figure of merit (FoM) summary, and prospect extension of GRM-based RRAM devices. With excellent physical and chemical advantages like intrinsic Young’s modulus (1.0 TPa), good tensile strength (130 GPa), excellent carrier mobility (2.0 × 10^5^ cm^2^∙V^−1^∙s^−1^), and high thermal (5000 Wm^−1^∙K^−1^) and superior electrical conductivity (1.0 × 10^6^ S∙m^−1^), GRMs can act as electrodes and resistive switching media in RRAM devices. In addition, the GRM-based interface between electrode and dielectric can have an effect on atomic diffusion limitation in dielectric and surface effect suppression. Immense amounts of concrete research indicate that GRMs might play a significant role in promoting the large-scale commercialization possibility of RRAM devices.

## 1. Introduction

**Figure 1:**
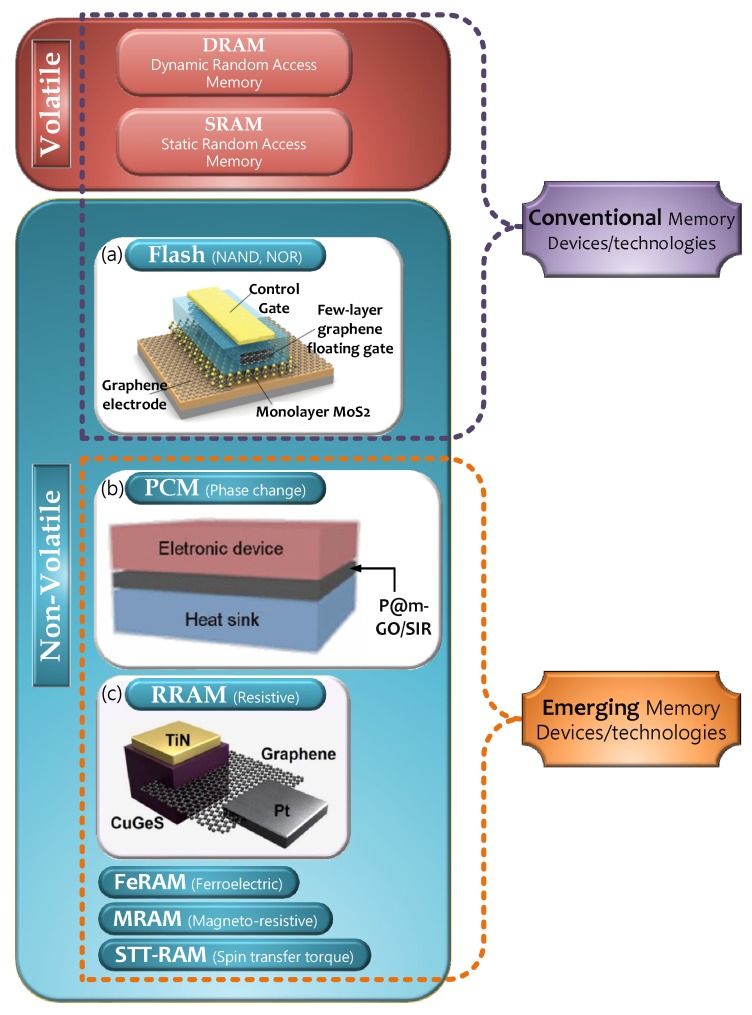
Diagram of conventional and emerging memory devices/technologies, including (**a**) a schematic illustration of flash memory device fabricated with graphene/MoS_2_ materials [2], (**b**) a schematic representation showing memory device with phase change materials P@m-GO/SIR [30], and (**c**) a schematic of graphene-based resistive random access memory (RRAM) device [31].

With the popularization of information technologies, digital problems with increasing amounts of data are receiving considerable attention in the industry. Until 2020, the digital world is expected to be over 40 zettabytes (i.e., 44 trillion gigabytes) with continuous expansion [1]. The rapid growth of computer processors, consumer electronic devices, and information exchange capacity calls for exponentially increased global data storage capability, which presents a high demand for advanced digital memory devices [1,2]. To deal with a huge amount of data, traditional memory devices based on von Neumann architecture are considered to be inadequate in the coming artificial intelligence (AI) era [3,4]. Conventional flash memory devices are facing a series of challenges including slow read and write (R&W) speed, limited device density, and high operational voltage [4,5,6,7]. As one of the most promising next-generation memory devices and one of the symbols of modern semiconductor technology, emerging non-volatile memory (NVM) devices are offering enhanced performance in terms of simpler structure, lower power energy consumption, reduced data access time, possibility of multiple states, and three-dimensional (3D) integration feasibility [6,7,8,9,10,11]. The technology report from Luigi Colombo presented the significance of emerging/prototypical NVM technologies [1], as illustrated in Figure 1, including phase-change memory (PCM), magnetic random access memory (MRAM), and resistive random access memory (RRAM). For now, some new NVM products were proposed by several companies such as carbon-nanotube RAM (NRAM) [12,13] and spin-transfer torque (STT)-RAM [14,15]. In the field of future high-performance computers and mobile electronic devices, these emerging NVM devices can work with operation speeds comparable to dynamic random access memory (DRAM) and are expected to replace static random access memory (SRAM) [12,13,14], which would provide far-reaching motivation to the development process toward so-called universal memory [12,13,14,15]. This denotes a single information-storage technology that can combine the best properties of data storage with hardware of memories, eliminating the need for multiple memory hierarchies within the same computing system. 

Currently, with the rapid development of solid-state devices and their excellent combination with emerging NVM technologies, impressive achievements of emerging NVM devices such as FeRAM, PCM, MRAM, RRAM, and STT-RAM were made due to the continuous improvement of fabrication processes, as well as through the performance of enhanced devices stimulated by the application of new materials at the nanoscale [6,7,8,9,10,11,12,13,14,15]. The relationship between the performance of NVM devices and a variety of materials is under investigation, which is used to improve the figures of merit (FOMs) of NVM devices. A series of nanoscale materials attracted tremendous attention for their different characteristics, including binary transition metal oxides (AlO*_x_*, TiO*_x_*, TaO*_x_*, and NiO_x_) [16,17,18,19], perovskite materials [20], chalcogenides (MoS_2_ and WS_2_) [21,22], organic materials [23,24], and graphene-based materials (graphene and graphene oxides) [25,26,27]. In addition to their atomic-scale thickness, some unique chemical and physical properties exist, such as the flexibility and transparency of these materials, which are highly desirable for the development of information-storage devices to be integrated in wearable systems and smart devices [28,29]. In the last few years, a growing attention was directed to carbon-based memristive devices, especially those based on graphene and graphene oxide (GO), due to the unique properties of this class of materials. 

The aim of this work is to provide a comprehensive overview of the state of the most significant advancements of graphene-based memristive devices. A brief introduction of the basic properties and synthesis methodologies of graphene-based materials (mainly graphene and GO) is presented in Section 2. In Section 3, an overview of graphene-based NVM devices (mainly memristors) is given with electrical performance, indicating essential characteristics that should define a working device. In Section 4, the main switching mechanisms based on oxygen ion diffusion and metal ion diffusion are demonstrated. In Section 5, a discussion of graphene-based memristors applied in flexible electronic devices is presented. 

## 2. Graphene-Based Materials: Properties and Production 

### 2.1. Properties of Graphene-Based Materials

During the fabrication and operation processes of electronic devices, the inherent properties of available materials receive paramount attention [19,32]. As one of the fundamental composition blocks of graphitic materials in all dimensionalities, graphene is a representative flat monolayer of carbon atoms tightly packed into a two-dimensional (2D) honeycomb lattice [33]. As reported by Geim et al., wrapped-type zero-dimensional (0D) fullerenes, rolled-type one-dimensional (1D) nanotubes, and stacked-type 3D graphite are external variations of graphene. Since the research started about 60 years ago, graphene was used extensively to explain the properties of various carbon-based materials [33]. Graphene is receiving considerable attention due to its special phenomena of predictability and measurability, mainly originating from the massless relativistic particle behavior of electrons [33]. The characteristics of graphene materials including intrinsic Young’s modulus (1.0 TPa) and tensile strength (130 GPa) [34,35], excellent carrier mobility at room temperature under ambient air conditions (2.0 × 10^5^ cm^2^∙V^−1^∙s^−1^) [36], and high thermal (5000 Wm^−1^∙K^−1^) and electrical conductivity (1.0 × 10^6^ S∙m^−1^) [15,37] make this material applicable as an active material in a variety of interdisciplinary fields such as supercapacitors [38,39], sensors [40,41], energy storage devices [42,43], and multifunctional fillers in nanocomposite materials [44]. 

Apart from the properties of graphene and cost consideration, memory devices developed with high-quality materials, a sophisticated nano-fabrication process, and sufficient prediction may demonstrate enhanced performance. However, in practice, a tradeoff among device performance, nano-scale fabrication process, and suitable cost is necessary, which indicates that it is essential to investigate novel memories that are adaptable to modern electronics. As the most representative category among 2D materials, graphene and related materials (GRMs) are expected to promising materials for the development of modern NVM devices due to their intriguing physical and chemical properties. For instance, the high mobility of graphene may contribute to faster operation speeds and lower operation times for NVM devices; the atomically thick structure of graphene can be helpful to realize the compact integration density of single devices; the solution-processable compatibility of graphene improves the successful probability of flexible memory devices with lower cost and a simpler fabrication method. Based on these considerations, GRM application for NVM devices with properties of higher density, faster operation speed, and lower energy consumption is receiving increasing interest. Hong et al. demonstrated a silicon (Si)-based flash memory device with graphene as a floating gate, which presented a wide operation window and low cell-to-cell interference with low operation voltage [45]. Qi and Shen et al. reported an RRAM device with a solution-processed GO thin film, which operated with an operation voltage lower than 2 V and a ~10^3^ on/off ratio [19]. These results suggest the great potential of GRMs in the NVM industry.

### 2.2. Synthesis Technologies of Graphene-Based Materials

It is over 10 years since Geim and Novoselov firstly found a way to fabricate graphite thin flakes, and several processes for the synthesis of graphene-based materials are mainly investigated using thin films. In general, it is easy to complete the deposition of a graphene monolayer onto a metal substrate, and the progress of this approach demonstrates the great potential of large-area single crystals [25,46,47,48]. Growth techniques reported in the literature can be observed in Table 1, including chemical vapor deposition (CVD), atomic layer deposition (ALD), nucleation and growth, liquid-phase exfoliation (LPE), electrochemical exfoliation, and a solution process [6,49,50,51,52,53,54,55,56,57,58]. Apart from graphene-based materials, some researchers like Zhang et al. also focused on other 2D materials such as MoS_2_-based composites, which presented excellent performance in the application of memristive devices, similar to that using graphene-based materials [58].

As typical conventional deposition methods, CVD and ALD are not only used to grow graphene, as well as multicomponent heterostructures of II–VI, III–V, and oxide materials, but they are also applied to the production of large-scale hexagonal boron nitride (h-BN) and two-dimensional transition metal dichalcogenides (2D-TMD) [59,60]. Currently, high-quality graphene materials with monocrystalline and polycrystalline structures are only reported to be grown by CVD and thermal desorption of Si from SiC single crystals [61], and notable progress was achieved in the deposition of graphene on metals [30,62,63]. In addition, although the growth of h-BN and TMD materials is also investigated, the growth of large-area monolayers or few-layer single crystals of h-BN and TMDs is still a major challenge [64,65]. Apart from CVD and ALD, the nucleation and growth approach was also investigated, showing that graphene single crystals could be deposited on metals like copper (Cu) and lithium (Li) [53,66]. During the nucleation and growth process, edge functionalization for graphene growth on noncatalytic surfaces may be required due to a higher edge growth rate being observed in 2D, which can be observed in TMD materials [60]. The LPE method is a versatile technology which was used to exploit 2D materials such as graphene, TMD, and h-BN from substrates [67,68,69]; these materials can then be applied into the large-scale production of 2D material-based devices. LPE can also be used to fabricate 2D material inks in different solvents [70,71,72], which reveals its great potential in flexible device fabrication. The electrochemical exfoliation method reported by Feng, Müllen, and co-workers [1] demonstrated that electrochemically exfoliated graphite provides graphene flakes from one to three layers with a high yield of >80%, a high C/O ratio of ~12, a sheet resistance value of 4.8 kΩ∙m^−1^, and hole mobility of 233 cm^2^∙V^−1^∙s^−1^ for a single sheet [1]. Currently, solution process methods of graphene and GO materials also receive considerable attention, mainly including spin coating, dip coating, and drop casting [73,74,75,76,77,78]. A comparison of these approaches is shown in Table 2.

## 3. Memory Devices and Memristors Based on Graphene-Based Materials 

### 3.1. FoM (Figure of Merit) of Memory Devices

In order to produce memory devices with smaller cell size, faster operation speed, lower power consumption, and lower fabrication cost, an FoM assessment of memory devices is always the priority [79,80]. Apart from the performance measurement of devices under development, FoM comparisons with other state-of-the-art market products are also considered. In general, the FoM assessment of NVM devices commonly focuses on basic characteristics in terms of operation speed, reliability, power consumption, scalability, and cost (Table 3) [15,79,80,81,82,83,84,85,86,87,88,89].

#### 3.1.1. Operation Speed

The random-access time of a single-memory cell and the effective time of write/erase operation performance (latency) are used to determine the operation speed of memory devices. For now, high-performance volatile memory devices, such as SRAM and DRAM, are commonly used in processors, which perform short latencies (<100 ns), but are more expensive and occupy larger chip areas as compared to silicon flash memories [81,82]. Although some disadvantages including higher cost and lower density exist, emerging NVM technologies demonstrated an operation time comparable to current SRAM and DRAM [83,84]. 

#### 3.1.2. Reliability

Write endurance and data retention properties are considered as main characteristics of NVM device reliability. Write endurance is the quantized behavior of the anti-fatigue-degradation characteristic of a single NVM device, which is determined as the highest number of write/erase cycles that can be operated before the NVM cell fails to be reliable. The endurance also demonstrates the number of sustained cycles of the device before reaching the breakdown state [15,85]. Data retention refers to the amount of time for which the information can be sustained within the NVM cell, which demonstrates the sustained time during which the device can keep the internal resistance states without external power [86,87]. A higher number of endurance cycles and a longer retention time are essential to maintain reliability in the device’s lifetime, which can avoid device failure and maintain device readability.

#### 3.1.3. Power Consumption

Device power consumption is made up of static power dissipation and dynamic power consumption. Static power dissipation mainly results from the aging process of storage hardware, and the main source of dynamic power consumption is memory data transition [90,91,92,93]. Compared with conventional silicon-based memory, memory devices with emerging memory technologies, including RRAM, PCM, FeRAM, and STT-MRAM, demonstrate less power consumption. In addition, these emerging memory devices show stronger resistance against the research trend of device miniaturization [94,95], which is applied to help data centers deal with their ever-increasing power requirement. 

#### 3.1.4. Scalability

In order to achieve a memory device with excellent performance, the scalability of the memory cell is receiving more and more attention due to the increasing density of memory devices, which indicates that the memory cell must be scaled. Apart from processing problems introduced by device scaling, another limitation factor, i.e., cross-talk between neighboring cells, cannot be neglected [20,96]. Typical values of bit density for state-of-the-art market products are expressed as the number of GB per chip together with the corresponding cell size expressed as multiples of F^2^ (F is the technology feature size). For now, the novel program of a semiconductor product with over 64 stacking layers of memory was realized on one microchip, which presents a high bit density of 0.5 GB/mm^2^ [97,98], which was considered as an achievement with record-high bit density. 

#### 3.1.5. Cost

In order to determine the commercial feasibility of a product program, during the manufacture of one memory chip with NVM technology, the cost of related materials, the fabrication process, and the integrated system should receive enough consideration. With the development of future semiconductors in the industry, the acceptable cost of a memory device will depend on target applications in terms of embedded hardware design, low-power-consumption environment, high speed, and high capacitor demand [99,100]. 

### 3.2. Memristor 

As one of the most promising non-volatile memory devices, the memristor demonstrates great potential in terms of enhanced reliability, fast operation speed, lower power consumption, and lower market cost, which can be observed in Table 3. In 1971, the theoretical concept of the memristor was proposed by Chua, which eluded the attention of integrated circuit designers as a single-device electronic implementation for the past three decades. With Chua’s perspective, all 2D NVM devices based on resistance switching performance are memristors, regardless of the device materials and physical operating mechanisms [101,102,103,104]. Researchers from Hewlett-Packard (HP) Laboratory firstly reported that they developed high-density non-volatile memories with a crossbar-based structure [105]. With a combination of complementary metal-oxide-semiconductor (COMS) technology and nanoscale-level processed memristors, profound influence will be observed on not only the flash memory industry but also the computing process of digital and neuromorphic systems. The memristor device exhibits a dynamical resistance state determined by its excitation history, which is used to build transistor-less nonvolatile semiconductor memory (NVSM), commonly known as RRAM [101,102,103,104,105,106].

Chua proposed the existence of another two-terminal component in the circuit, in addition to the resistor, capacitor, and inductor, which operated on the basis of a relationship among voltage (*ν*), current (*i*), magnetic flux (*φ*), and charge (*q*) [107]. The resistor is used to describe the relationship between *ν* and *i*, the capacitor is the connection between *ν* and *q*, and the inductor links *φ* and *i*. Therefore, in general, the memristor can be used to define the connection between *φ* and *q*. To be more precise, the definition of a memristor is related to not only the flux as a magnetic magnitude but also the mathematical magnitude corresponding to the time-integral of the voltage across the device [101,102,104,106]. The most obvious characteristic of a memristor is that its resistance changes with the amount of current passing through the device. The resistance will stay at the previous state even if the passing current stops. Until the device receives reverse current, the resistance state will change. 

With the development of memristor research for about half a century, RRAM devices received considerable attention as the most typical memristor. Apart from research on device performance, recent research was directed toward the study of materials with resistive switching (RS) function, such as binary transition metal oxides (TiO_x_, AlO_x_, and NiO_x_) [3,107,108,109,110,111], perovskite compounds (CH_3_NH_3_PbI_3_ and CsPbBr_3_) [54,55,112], ferromagnetic materials [112,113], biological materials [114,115], and graphene-based materials (graphene and GO) [30,116]. 

#### 3.2.1. RRAM Device Characteristics

**Figure 2:**
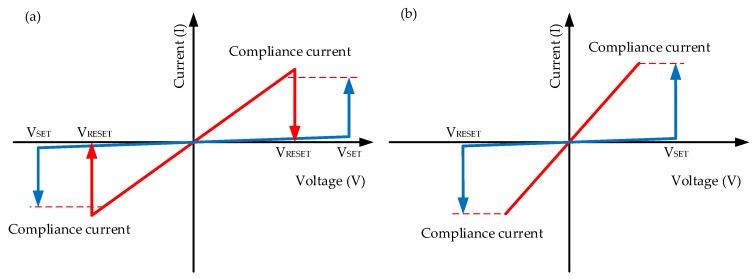
Unipolar (**a**) and bipolar (**b**) resistive switching (RS) behavior of RRAM device [3,107,108,109,110,111].

RRAM devices fabricated with a typical MIM (metal–insulator–metal) sandwich structure are electrically characterized with two different resistance states, which are generally referred to as the high-resistance state (HRS) and low-resistance state (LRS). The high-resistance value of the device shows the low-conductance state, while the device demonstrates the high-conductance state with the low-resistance value. The on/off ratio is determined by the ratio between HRS and LRS. With the applied voltage bias, the set operation is defined from HRS to LRS, and the reset operation is the transition from LRS to HRS. Stop voltages of the set and reset process are defined as V_SET_ and V_RESET_. In general, two different switching types are defined as unipolar and bipolar [3,107,108,109,110,111], as illustrated in Figure 2. The unipolar switching mode is defined by the amplitude of the applied voltage bias, while the bipolar switching depends on the polarity of the applied voltage bias. In addition, as an NVM device, the endurance and retention properties of RRAM are also the basis of device reliability. 

#### 3.2.2. RRAM Based on Graphene and Its Derivatives

**Figure 3:**
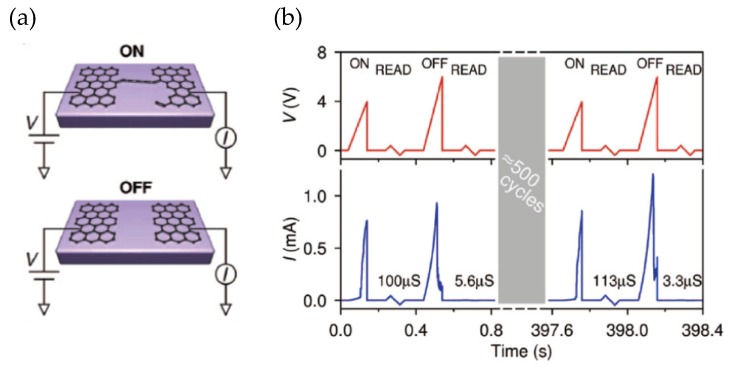
(**a**) Proposed schematic atomic configurations in the on and off states of transistor-type memory. (**b**) Repeatable programming over hundreds of cycles [119].

In general, graphene and its derivatives are investigated as resistive switching (RS) and electrode materials in RRAM devices. Exfoliated graphene sheets with RS performance in a field-effect transistor (FET) were reported by Tsang and Delgado-Notario et al. [117,118,119]. The external electrical field of the gate electrode with the presence of OH^−^ and H+ absorbed on the SiO_2_/Si substrate induced a chemical modification in the crystal structure of graphene. Bruck et al. reported a graphene-dielectric-layer-based RRAM device with an on/off ratio of ~100 and switching time of ~100 us in 2008, as illustrated in Figure 3 [119]. After that, an RRAM cell fabricated with graphitic stripe layers was reported by Li et al., which demonstrated a higher ratio (~10^7^) and faster switching speed (~1 us) [1]. For now, as one of the most representative graphene derivatives, GO receives more consideration due to its similar flexibility and robustness to graphene via a simple synthesis method and its enhanced RS performance as a dielectric medium [120]. GO can be fabricated via the oxidation and exfoliation of graphite before being dispersed in water; the GO suspension can be easily deposited on the bottom electrode (BE) via various methods including spin coating, drop coating, and vacuum filtration [121,122,123]. Following a thermal annealing process, the GO thin film can be fabricated after a suspension deposition process. In general, the annealing temperature is commonly lower than 200 °C, and the thickness range of obtained GO thin films is from about a few tens of nanometers (nm) to a few tens of micrometers (μm) [19,121,124,125,126,127,128]. 

In addition, various groups investigated graphene-based composite materials, such as combinations of GO/rGO (reduced GO) with polymers, small molecules, and metal nanoparticles (NPs). An Al/TPAPAM-GO/ITO device was proposed by Zhuang et al. [129]. An arylamine-containing conjugated polymer TPAPAM was chosen as a hole-transport agent due to its excellent hole injection, high mobility, and low ionization potential. TPAPAM-GO was fabricated using soluble triphenylamine-based polyazomethine (TPAPAM), which was used to covalently graft GO and form TPAPAM-GO. TPAPAM-GO-based RRAM devices demonstrated typical bipolar RS performance with an on/off ratio of ~10^3^ at ~1 V operation voltage and rewritable memory performance with stable resistance states for more than 10^8^ s [129]. Jin et al. [130] reported a device with a ferrocenylphenyl-NHCO-GO (FPArGO) RS layer synthesized from ferrocene and GO. Ferrocene is a small molecule with 18 π-electrons and a redox-active organometallic compound at low potential. They synthesized FPArGO as a conjugated graphene-based material with redox active small molecules covalently bonded to an active layer of rGO. Enhanced RS performance of Al/FPArGO/ITO RRAM devices can be observed with an operation voltage lower than ~2 V and an on/off ratio around ~10^3^. The write and read cycles could be repeatedly performed in excess of 1000 cycles, and the data sustainment time was beyond 10^4^ s [130]. Cui et al. [130] proposed nonvolatile memory devices with Au nanoparticles (AuNPs) and reduced GO (rGO) sheets in both horizontal and vertical structures. Additionally, 4-mercapto-benzenediazonium tetrafluoroborate (MBDT) salt was used as a π-conjugated bifunctional molecular bridge to chemically bind AuNPs to the rGO channel, which created functionalized rGO (frGO). Al/AuNPS-frGO/ITO memory devices exhibited bipolar IV characteristics with an operation voltage lower than ~3 V, an on/off ration higher than ~10^3^, and retention time sustained over 700 s [130]. In general, compared with memory devices with pure GO materials, a GO layer combined with other compound materials can exhibit enhanced RS performance.

**Figure 4:**
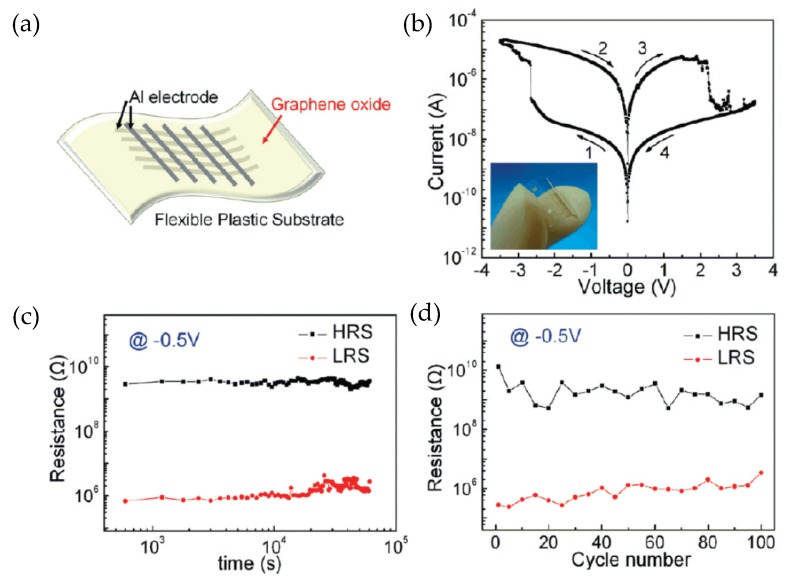
(**a**) Schematic illustration of Al/GO/Al/PES flexible crossbar memory device; (**b**) Typical I–V characteristic of Al/GO/Al/PES RRAM device; (**c**) Retention property of Al/GO/Al/PES device read at −0.5 V; (**d**) Endurance performance of Al/GO/Al/PES device measured during 100 sweep cycles [124].

For RRAM devices with a GO-based MIM structure, the choice of top electrode (TE) and bottom electrode (BE) materials always receives the most attention. Table 4 exhibits various TE and BE combinations of GO-based RRAM devices. It is suggested that the RS performance of GO-based RRAM devices is mainly related to the different work functions (ф_M_) of TE and BE [120,121,131]. In Table 4, Au, Al, Ag, Cu, and Ti are always used as the TE while Pt is commonly used as the BE in electronic devices. The ф_M_ of Pt is ~5.65 eV, while the ф_M_ of Au, Ti, Ag, and Cu is ~5.1 eV, ~4.33 eV, ~4.26 eV, and ~4.65 eV, respectively. The existence of a work function difference (Δф_M_) between TE and BE, namely, an asymmetric work function between active and inert electrode, results in the appearance of RS performance in these GO-based RRAM devices [120,121,122,123,124,131,132]. For GO-based devices with ITO (indium tin oxide) as the BE, the ф_M_ of ITO is ~4.9 eV, which is very close to the ф_M_ of metal Au. Therefore, no effective RS performance can be observed in Au/GO/ITO structure devices [124,133]. It is worth noting that devices with Al (~4.28 eV) as the TE show unusual performance, in contrast to devices with other TE metals. Although the Δф_M_ between Al and Pt is higher than 1.0 eV, successful RS behavior cannot be observed in Al/GO/Pt RRAM devices. However, as illustrated in Figure 4, Al/GO/Al RRAM devices with no Δф_M_ exhibit enhanced RS performance. Compared with metals like Au, Ag, and Cu, local oxidation is more likely to occur on Al electrodes at the interface, which might be a decisive influence factor explaining its unique performance [120,121,122,123,124,131,132,133]. As reported by Liu et al. [8], the work function difference between TE and BE metals has significant effects on device characteristics and performance. RRAM devices made from TE and BE metals with larger work function difference have a higher and more stable on/off resistance ratio with larger set and reset voltages.

**Figure 5:**
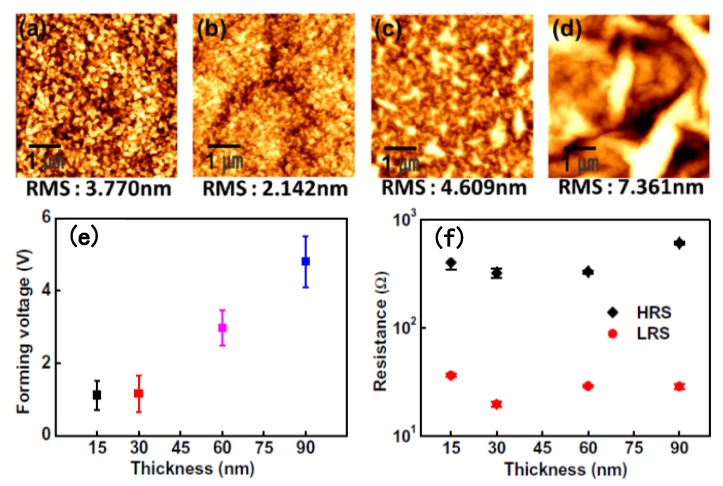
The surface roughness of GO after deposition on various bottom electrodes: (**a**) ITO, (**b**) Pt, (**c**) Al, and (**d**) Au. (**e**) GO film thickness dependence of forming voltage of Cu/GO/Pt memory cells. (**f**) High- and low-resistance states (HRS and LRS) of Cu/GO/Pt memory cells, plotted against the thickness of GO [132,133].

Apart from the choice of TE and BE, other features like surface roughness and obtained thickness of the GO dielectric layer cannot be neglected, as illustrated in Figure 5 [132,133]. Figure 5a–d demonstrate the surface roughness of a GO thin film deposited on different BEs, observed using an atomic force microscope (AFM). The low surface roughness of GO thin films can be obtained with its deposition on a smooth BE, such as inert metals like Pt (RMS 2.142 nm) and compounds like ITO (RMS 3.770 nm). Higher roughness is observed for GO thin films with BEs like TaN and Au due to the existence of cracks. Because of the presence of these cracks and a rough surface in GO dielectric layers, penetration might occur more easily in electrode materials, leading to failure of effective RS performance. Device discrepancy likely results from contrasting thermal properties between GO and electrode materials. As illustrated in Figure 5e, the forming voltages of Cu/GO/Pt RRAM devices are obviously influenced by the thickness of GO films. During the forming process, the required voltages synchronously increase with the thickness of GO thin films, which indicates that the internal electrical field in the GO dielectric layer plays a decisive role in the forming operation. After the forming operation, Figure 5f demonstrates the resistance states (HRS and LRS) of Cu/GO/Pt RRAM devices. The relationship between resistance values and thickness of GO thin films can be considered as a function, as reported by Zhuge et al. [132]. 

## 4. Switching Mechanism of Graphene-Based Memristor

### 4.1. Oxygen Ion-Based Switching Mechanism

**Figure 6:**
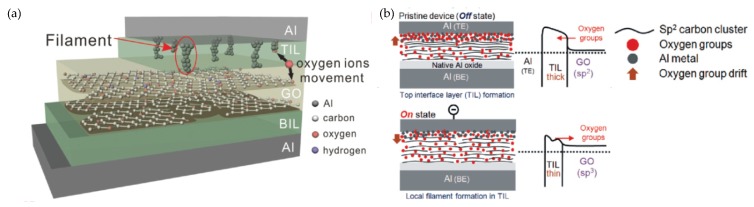
(**a**) Schematic of oxygen ion movement model of Al/GO/Al RRAM device. (**b**) Schematic of the proposed bipolar RS model for Al/GO/Al crossbar memory device [124,143].

Several studies reported that the main reason for RS performance in dielectric layers based on graphene and its derivatives, especially GO layers, is the diffusion of oxygen ions or vacancies [19,134,135,136,137,138,139]. In general, the RS behavior and switching mechanism are influenced by the fabrication methods and hybridization state of graphene-based material dielectric layers, the deposition techniques of electrode layers, and the choice of TE and BE materials. Some studies agreed that the structure modification of the entire dielectric layer results in a comprehensive effect on RS behavior [123,131,140]. However, other studies considered that the RS behavior is a regional phenomenon, as illustrated in Figure 6a, which is associated with the contact resistance between the TE and dielectric layer [22,38,141,142,143]. 

In 2010, as one of the first examples focused on the oxygen ion-based switching mechanism of graphene-based memory devices, Kim et al. proposed Al/GO/Al crossbar memory on flexible substrate [124]. As illustrated in Figure 6b, due to the redox reaction process occurring at the interface between GO and the Al electrode, the resulting interface layer plays a decisive role in the switching process. This AlO*_x_* layer acts as an insulating layer and influences the HRS. With the applied negative voltage bias, electrical-field-induced oxygen ions diffuse into the GO layer, which forms local conductive filaments (CFs), resulting in the device switching into the LRS. However, ohmic conduction did not occur at the LRS due to the GO film transforming into an *sp*^3^-bonded state without CFs [124].

One year later, Panin et al. also reported a device with an Al/GO/Al structure, which presented the importance of local resistance state induced by the contact of Al and GO layers [144]. With the positive voltage bias applied onto the Al layer in the forming process, a highly resistive region formed near the TE. Under the effect of an external electrical field, oxygen ions stored in the dielectric layer drift to the electrode, which can result in the continuous formation of an *sp*^3^ hybridization layer between Al and the structure-modified GO layers at the HRS. When a negative voltage bias is applied onto the TE Al layer, the reverse diffusion of oxygen ions leads to LRS CFs near the contact interface under the effect of a negative electrical field [144]. 

**Figure 7:**
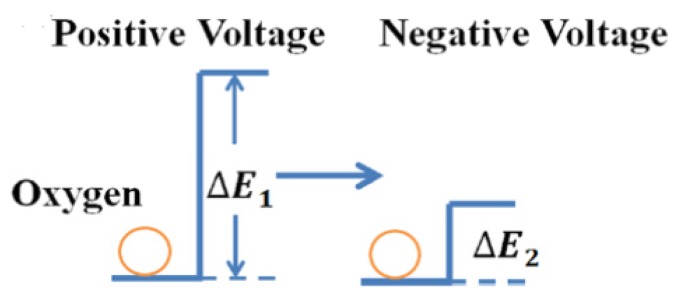
Schematic of oxygen hopping barrier change model for Al/GO/ITO RRAM devices [145].

According to a report from Wang et al. [145], the diffusion of oxygen ions can be used to explain the switching performance, as well as the operation speed, of graphene-based RRAM devices. The oxygen ion migration of a device is affected by the existence of a barrier on hopping energy. The inhomogeneity of electron density in a dielectric layer is a decisive influence factor in the electron extraction and injection process, which significantly affects the performance of the hopping energy barrier. During the set operation, the hopping energy barrier is reduced due to the electron extraction of the dielectric layer, which increases the oxygen ion mobility, as illustrated in Figure 7. Conversely, the electron injection process from the metal electrode into the dielectric layer during the reset operation increases the energy barrier and decreases the diffusivity of oxygen ions. Therefore, compared with its long set speed (~80 us), the RRAM device in Reference [145] showed a shorter speed (~90 ns) in the reset operation. 

### 4.2. Metal Filament-Based Switching Mechanism

**Figure 8:**
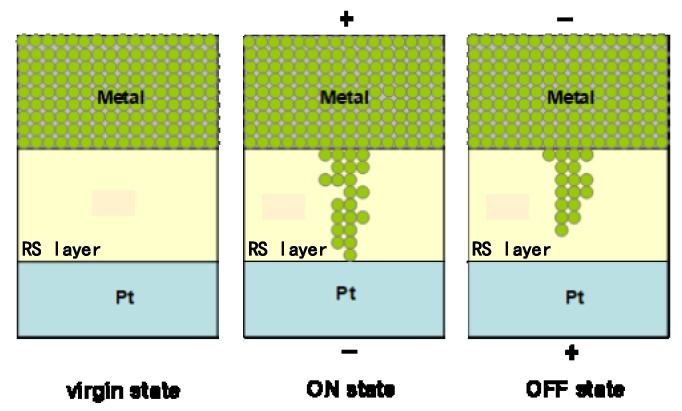
Schematic diagram for the mechanism of the resistive switching effect in metal/GO/Pt memory cells [132].

Apart from the switching mechanism based on oxygen ion diffusion, a metal filament-based mechanism was also used to explain the RS behavior of graphene-based RRAM devices, which mainly depends on electrode materials [99,132,133,146,147]. Zhuge et al. reported this mechanism in a metal/GO/Pt structure device, as illustrated in Figure 8 [132]. In this case, several metals such as Ag, Ti, Cu, and Au were used as the TE in the devices. They inferred that the low switching voltage was related to the ion diffusion coefficient of electrode materials. Metal/GO/Pt devices with an active TE like Ag, Ti, and Cu with a large ion diffusion coefficient were more likely to be operated with low forming voltage. Au/GO/Pt devices which worked with higher forming voltage resulted from gold being very difficult to oxidize to ions [132]. During the set operation, the external electrical field induced by a positive voltage bias on the TE resulted in the metal generating ions in the graphene-based dielectric layer, thereby forming the CF paths, which caused the device to be in the on state that was maintained until the presence of a negative voltage bias. After the TE experienced a negative voltage bias, the metal-based CF paths were dissolved by the electrochemical effect between the metal and dielectric layer, which caused device to be in the off state [132,133]. 

**Figure 9:**
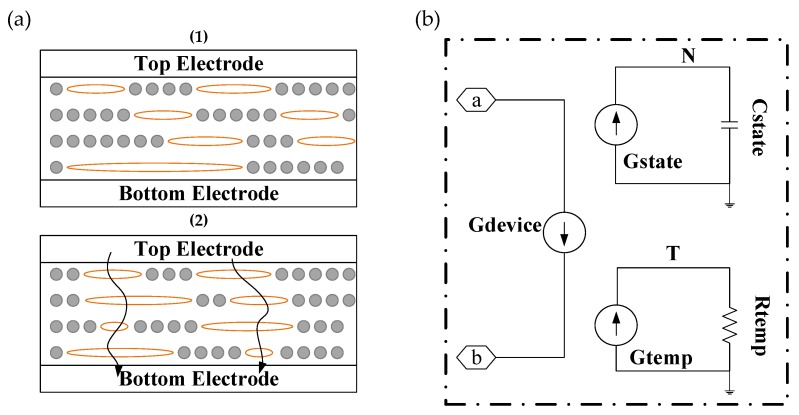
(**a**) Illustration of transport mechanism in GO-RRAM based on hybridization state modulation; (**b**) SPICE macro implementation of the compact hybridization state modulation model.

In addition to the two main switching mechanisms discussed above, a mechanism affected by GO-based bulk was also investigated. It is worth mentioning that Wah et al. presented a compact model of hybridization state modulation for GO-RRAM, which is one of the most effective explanations for the bulk mechanism [28,139,148,149,150]. The proposed model associates the resistance switching mechanism with the electrical-driven modification of *sp*^2^ cluster density in the switching layer. A simulation program with integrated circuit emphasis (SPICE) model incorporating the proposed compact model was developed for parameter calibration. Firstly, they provided the transport mechanism in GO-RRAM based on hybridization state modulation, as illustrated in Figure 9a. The amorphous *sp*^3^ matrix isolates these graphene-like clusters, which introduces a high tunneling barrier and substantially limits the electron tunneling probability between the *sp*^2^ clusters. The initial resistance of GO-RRAM is determined by the *sp*^2^ clusters. The increasing *sp*^2^ density induced by oxygen functional group reduction results in hopping paths for the electron, which completes the set operation (HRS to LRS) [149,151]. Conversely, with a positive voltage bias, oxygen ions drift back to the GO layer, while *sp*^2^ clusters are hybridized into *sp*^3^ bonding, which reduces the *sp*^2^ density and switches the device back into the HRS. Acting like intermediate traps, *sp*^2^ clusters support continuous electron flow, which can be explored via a multi-phonon trap-assisted tunneling (MTAT) mechanism [151]. Figure 9b exhibited the SPICE macro implementation of the proposed compact model, as well as a comparison of I–V characteristics of the SPICE simulation and experimental result. The I–V characteristic of the proposed compact model has high correlation with the experimental data [148,151]. A similar discussion mechanism of bulk GO was also supported by Romero et al. [139], whose work mainly focused on different switching mechanisms of GO memristive devices.

**Figure 10:**
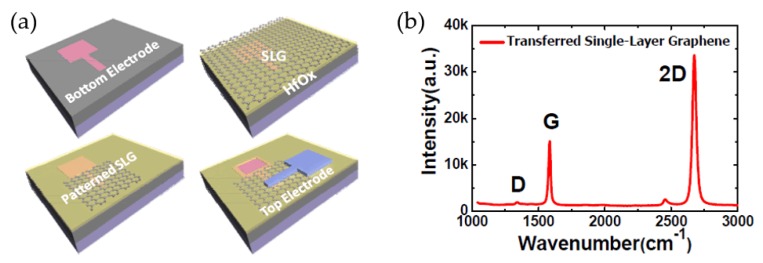
(**a**) Fabrication process of HfO*_x_*-based RRAM device with single-layer graphene (SLG) interlayer and (**b**) Raman spectroscopy data of transferred SLG layer [152].

In addition, some other research teams aimed at an investigation of the contacting relationship between the GRM layer and electrode layer or other RS layers, mainly related to single-layer graphene (SLG) or multi-layer graphene (MLG) interlayers applied in RRAM devices with metal–oxide (M–O) thin films [139,152,153,154,155,156]. In the literature review, it is noteworthy that Chen et al. presented a TiN/SLG/HfO_2_/Pt RRAM device with an SLG interlayer [152]. The HfO_2_ layer was fabricated by ALD, and the SLG layer was transferred onto the RS layer with oxygen plasma etching. The inserted SLG layer increased the resistance value at LRS (>1 MΩ) of the HfO_2_-based RRAM device due to its high out-of-plane resistance, which reduced the reset current 22-fold while decreasing the programming power consumption 47-fold. With the measurement results from Raman mapping illustrated in Figure 10b, obvious changes in the SLG interlayer in both the D-band and the G-band signals can be observed, which indicate that oxygen drifting from the M–O layer might interact with the graphene. Chen’s work also illustrated that the interface engineering design is very essential for the selection of RS and electrode materials for RRAM devices.

## 5. Application of Graphene-Based Memristors in Flexible Electronics

As one of most representative and competitive graphene-based materials, GO received considerable attention in the industry of large-area flexible electronic devices due to its promising potential for next-generation information storage [156,157,158,159,160,161,162,163]. GO can be dispersed in a variety of solvents, which permits compatible processes with a wide range of commercially available flexible substrates. With various technologies like spin coating [164,165,166], drop casting [167,168,169], dip coating [170,171,172], and inkjet printing [157,173,174], GO dielectric layers can be deposited onto flexible substrates like polyethylene terephthalate (PET), polyether sulfone (PES), and polyimide [175,176,177,178,179,180]. In addition, the thickness of a single atomic layer and the excellent dispersibility in various solvents result in GO having enhanced compatibility with different commercial substrates [181,182,183]. Table 5 demonstrates several samples of graphene-based memristive devices with electrical performance.

According to the statistical results from Table 5, the most popular RS medium of flexible GRM-based RRAM devices is GO, whereas Al and ITO are considered preferable for the role of TE and BE, respectively. The structure of an Al/GO/ITO/flexible substrate is even more popular. 

**Figure 11:**
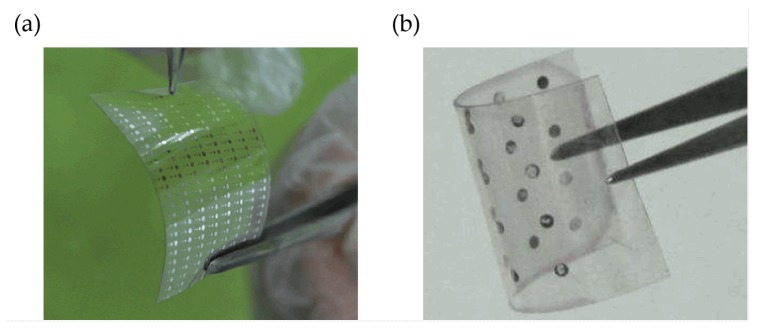
Illustration of GO-based flexible RRAM devices with structures of (**a**) Al/GO/ITO/PET and (**b**) Al/Au/rGO/PVA NP/ITO/PET [148].

Wang et al. reported a GO-RRAM device with the structure of Al/GO/ITO/PET, as illustrated in Figure 11a. The GO thin film was spin-coated onto the ITO substrate. According to their perspective, the speed of rotation process and the GO precursor solution concentration determined the growth rate and thickness of the GO thin film. They controlled the growth rate at around 0.4 nm per GO drop, and the rotation speed was 1000 rpm. The fabricated device could operate with an operation voltage lower than ~2.5 V and the on/off ratio was about ~280. The retention was higher than 10^4^ s, and the endurance cycles were more than 110. In addition, with the pulse behavior measurement, they also found that the resistance state switching from LRS to HRS could appear with a 100-ns pulse width, while the switching process from HRS to LRS could only be achieved with a 100-μs pulse width [145]. 

Another RRAM device based on an Al/rGO/ITO/flexible substrate with a combination of Au nanoparticles (Au NPs) and polyvinyl alcohol (PVA) was reported by Midya et al. The Au/rGO/PVA dielectric layer was fabricated with a solution-processed method. In this work, in order to prevent Au NP coagulation, GO flakes fabricated via a modified Hummer’s method and HAuCl_4_ solution were homogenized into the PVA solution. With a series of chemical treatments, the hybrid thin film was spin-coated onto the ITO/PET substrate. The fabricated device demonstrated a bipolar RS performance with ~1-V operation voltage in the SET process and a ~10^3^ on/off ratio. The resistance distribution result indicated that the device exhibited better performance when the resistance state switched to LRS [185]. 

Apart from Wang and Midya et al., Hong et al. also reported a GO-RRAM device with the same structure, which was a transparent Al/GO/ITO device with a flexible PET substrate. Different GO dielectric layers with three thicknesses (~15, ~30, and ~45 nm) were also fabricated with a spin-coating process, and the device exhibited enhanced performance with a high on/off ratio, low set/reset voltage, and excellent data retention. With the statistical results of GO thickness dependence on fabricated devices, an RRAM device with a 30-nm GO layer exhibited the lowest operation voltage (~2 V) and the highest on/off ratio (~10^3^), which indicated that the 30-nm-thick GO thin film might be an optimistic choice for their investigation. It is noted that the fabricated flexible GO memory device with 30 nm thickness demonstrated excellent RS performance with no degradation, even when the substrate was bent down to 4-mm radius 1000 times [121].

Although the Al/GO/ITO structure received substantial attention, another kind of structure with Al/GO/Al on a flexible substrate was also investigated. Our previous work demonstrated an Al/GO/Si/Al structure RRAM device with a spin-coated GO dielectric layer. The device could operate with an operation voltage lower than 2 V, and the on/off ratio was around 10^3^. The retention performance indicated that the device could sustain data over 10^4^ s, and its endurance cycles exceeded 100. The GO suspension liquid of graphite oxide powder and ethyl alcohol was spin-coated onto the substrate, which indicates the great potential of a solution-processed GO dielectric layer to be deposited onto a flexible substrate [19].

**Figure 12:**
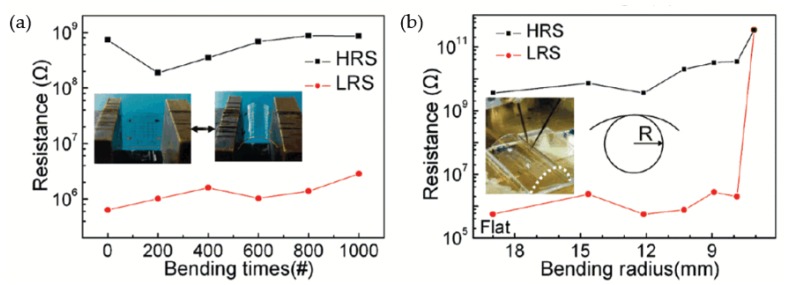
Measurement of bending effect for Al/GO/Al/PES RRAM devices. (**a**) The relationship between on/off ratio and bending times and (**b**) the relationship between on/off ratio and bending radius [124].

As illustrated in Figure 12, Jeong et al. reported an RRAM device with an Al/GO/Al/PES structure, which was fabricated with a spin-casted GO thin film. The fabricated device could complete the switching process with an operation voltage lower than 2 V and an on/off ratio higher than 10^3^. Figure 12b demonstrates the measurement effect of device performance induced by bending times and radius, which indicates that the Al/GO/Al/PES structure is suitable for flexible NVM devices. According to their perspective, the spin-casting process with enough simplicity and scalability can be a choice for the fabrication of large-area uniform GO thin films, whereas the final goal is to allow the fabricated GO thin film to be transferred to all kinds of flexible substrates. Furthermore, the spin-coating technology applied to practical devices might have great potential for integration with current standard COMS processes, in order to provide a contribution during the development of NVM devices [124]. 

Liu et al. illustrated their research on an Al/GO/Al/PET structure RRAM device on PET substrate with a spin-coated GO dielectric layer [163], which showed excellent RS performance with a low operation voltage (~2 V), high resistance ratio between on and off states (~10^5^), and long data retention time (~2000 s). The formation and the rupture of CFs based on metal ion diffusion near the interface layer between TE and dielectric layer were used to interpret their achievements [132,133,163].

Apart from GO materials, a mixture of graphene-based materials and 2D materials (like MoS_2_ and h-BN) was also investigated in recent years [188,189,190,191]. The surface modification of GO layers in solution through chemical functionalization is expected to play a key role in tailoring the structure, processability, and physicochemical and electronic properties of GO layers. As reported by Wu et al. [188], with vigorous stirring and spin-coating operations for a precursor suspension of MoS_2_ and GO, they fabricated an RRAM device with a Ti/MoS_2_-GO/ITO/PET structure. Due to the doping of MoS_2_, the device worked with a lower operation voltage (~0.3 V). Their bending test results showed that almost no changes in LRS and HRS could be observed during 200 switching cycles, even with 300 bending cycles, which indicated the enhanced reproducibility of RRAM devices [188]. The electron mobile induced by different energy levels of each layer was used to explain the switching mechanism [188]. 

## 6. Conclusions

We briefly reviewed recent advances in graphene-based materials used in NVM devices. Reliable systems based on memristors will be a vital technological application in the future development of neuromorphic networks and the AI industry, which are presented with a combined form of RRAM hardware devices and neuromorphic algorithms. Different devices based on graphene-materials mentioned in the recent literature exhibited good switching performance such as low power consumption, enhanced retention and endurance properties, large on/off ratio, and good compatibility with flexible substrates. Despite the great potential of graphene-based materials applied in NVM demonstrated in related research, some limitations cannot be neglected. Firstly, GO as the derivative of graphene was used extensively in electronic device research. The occurrence and organization of oxygen functionalities, the intrinsic variability of the GO material chemical structure, and the random distribution of GO flakes during the fabrication and deposition process directly influence the switching behavior of devices. Secondly, many current synthesis technologies of graphene-based materials are not only mature in the commercial market but also limit the development of large-scale production with good uniformity. Thirdly, the reliability performance of devices, in terms of retention and endurance measurements, needs to be evaluated and improved more accurately in order to meet real application demands in commercial products. In addition, although related switching mechanisms were proposed in the recent literature, definitive evidence in their support remains to be provided.

## Figures and Tables

**Table 1:** Comparison among main synthesis technologies and applications for graphene and its derivatives [6,49,50,51,52,53,54,55,56,57,58].

Synthesis Methods	Chemical Vapor Deposition	Atomic Layer Deposition	Nucleation and Growth	Liquid Phase Exfoliation	Electrochemical Exfoliation	Solution Deposition
**Materials**	Graphene	Graphene	Graphene	GO	GO	GO
	h-BN	h-BN	TMD	TMD	TMD	h-BN
	TMD	TMD				
**Devices**	FeRAM	FeRAM	MRAM	FeRAM	FeRAM	FeRAM
	MRAM	MRAM	PCM	RRAM	RRAM	RRAM
	TRAM	RRAM	RRAM	Flash	Flash	
	RRAM		STT-RAM			
	STT-RAM					

**Table 2:** Comparison among main solution-processed technologies [73,74,75,76,77,78].

Item	Spin Coating	Dip Coating	Drop Casting
**Fabrication cost**	Low	High	Low
**Fabrication equipment**	Spin coater, hot plates	Dip coater, hot plates	Hot plates
**Fabrication time**	<1 h	>2 h	<1 h
**Dielectric thickness**	Thin and uniform	Thick and uniform	Thick and heterogeneous
**Device performance**	Long retention time/endurance cycles	Short retention time/endurance cycles	Short retention time/endurance cycles

**Table 3:** Figure of merit (FoM) comparison among main conventional and emerging memory devices [15,79,80,81,82,83,84,85,86,87,88,89].

FoM	SRAM	DRAM	Flash NAND	RRAM	FeRAM	PCM	STT-MRAM
**Density (bit/chip)**	~10 MB	~10 GB	~10 GB	~1 GB	~1 MB	~10 GB	100 MB
**Technology feature size F (nm)**	16	15	15	16	65	20	22
**Cell size (F^2^)**	>100	~10	~5	~20	~40	~20	~40
**Operation speed (write time)**	~10 ns	~10 ns	~100 us	~100 ns	~100 ns		
**Program power/bit**	~10 pJ	~10 pJ	~10 nJ	~10 pJ	~1 pJ	~1 nJ	~1 pJ
**Retention time**	Volatile	Volatile	>10 years	>10 years	>10 years	>10 years	>10 years
**Endurance cycles**	~10^15^	~10^15^	10^5^	10^9^	10^15^	10^8^	10^15^
**Price ($/GB)**	<100k	~10	~1	~1k	~100k	~100	10k

**Table 4:** Comparison among GO-based RRAM devices with various electrodes [121,124,132,133].

TE	BE	RS Behavior	On/Off Ratio	V_Forming_ (V)	V_SET_ (V)	V_RESET_ (V)	Endurance (Cycle)	Retention (s)	Reference
Au	Pt	Bipolar	~60	~2.8	~0.7	~-0.6	>100	>10^5^	[132]
Ag	Pt	Bipolar	~60	~0.5	~0.5	~-0.3	>100	>10^5^	[132]
Cu	Pt	Bipolar	~15	~1.8	~0.5	~-0.6	>100	>10^4^	[132]
Ti	Pt	Bipolar	~10^3^	~1.2	~1.0	~-0.8	>100	>10^5^	[132]
Au	ITO	N.A.	N.A.	N.A.	N.A.	N.A.	N.A.	N.A.	[121,133]
Al	Al	Bipolar	~10^3^	N.A.	~2.0	~-2.5	>100	>10^5^	[124]
Al	ITO	Bipolar	~10^3^	N.A.	~2.0	~-1.5	>100	>10^9^	[133]
Al	Pt	N.A.	N.A.	N.A.	N.A.	N.A.	N.A.	N.A.	[132]

**Table 5:** Flexible memristive devices with GO-based materials [121,124,145,163,184,185,186,187].

Structure	Flexible Substrate	Fabrication Method of RS Layer	On/Off Ratio	Retention Time (s)	Endurance Cycles	Memory Type
Al/GO/ITO	PET	Spin coating	10^3^	10^7^	100	RRAM
Al/CMC-GO/Al	PET	Spin coating	10^5^	2000	N.A.	RRAM
Al/GO/Al	PES	Coating	200	10^4^	N.A.	RRAM
Al/GO/ITO	PET	Spin coating	280	10^4^	120	RRAM
Al/GO/Al	PES	Spin casting	10^3^	10^5^	100	RRAM
Al/Au-rGO/ITO	PET	Spin casting	10^3^	10^5^	N.A.	RRAM
hrGO/lrGO/hrGO	PET	Spin coating	10^3^	1000	N.A.	RRAM
Al/GO/ITO	PET	Drop casting	30	10^4^	N.A.	RRAM
